# Revolutionizing immune research with organoid-based co-culture and chip systems

**DOI:** 10.1093/cei/uxae004

**Published:** 2024-01-27

**Authors:** Diana Papp, Tamas Korcsmaros, Isabelle Hautefort

**Affiliations:** Department of Metabolism, Digestion and Reproduction, Imperial College London, London, UK; NIHR Imperial BRC Organoid Facility, Imperial College London, London, UK; Department of Metabolism, Digestion and Reproduction, Imperial College London, London, UK; NIHR Imperial BRC Organoid Facility, Imperial College London, London, UK; Food, Microbiome and Health Programme, Quadram Institute Bioscience, Norwich Research Park, Norwich, UK; Department of Metabolism, Digestion and Reproduction, Imperial College London, London, UK; NIHR Imperial BRC Organoid Facility, Imperial College London, London, UK; Food, Microbiome and Health Programme, Quadram Institute Bioscience, Norwich Research Park, Norwich, UK; Earlham Institute, Norwich Research Park, Norwich, UK

**Keywords:** organoids, immune cells, organ-on-chip, diseases modeling, drug screening, personalized medicine

## Abstract

The intertwined interactions various immune cells have with epithelial cells in our body require sophisticated experimental approaches to be studied. Due to the limitations of immortalized cell lines and animal models, there is an increasing demand for human *in vitro* model systems to investigate the microenvironment of immune cells in normal and in pathological conditions. Organoids, which are self-renewing, 3D cellular structures that are derived from stem cells, have started to provide gap-filling tissue modelling solutions. In this review, we first demonstrate with some of the available examples how organoid-based immune cell co-culture experiments can advance disease modelling of cancer, inflammatory bowel disease, and tissue regeneration. Then, we argue that to achieve both complexity and scale, organ-on-chip models combined with cutting-edge microfluidics-based technologies can provide more precise manipulation and readouts. Finally, we discuss how genome editing techniques and the use of patient-derived organoids and immune cells can improve disease modelling and facilitate precision medicine. To achieve maximum impact and efficiency, these efforts should be supported by novel infrastructures such as organoid biobanks, organoid facilities, as well as drug screening and host-microbe interaction testing platforms. All these together or in combination can allow researchers to shed more detailed, and often patient-specific, light on the crosstalk between immune cells and epithelial cells in health and disease.

## Teaser

Potential use and translational applications of patient-derived organoids are very diverse. Such models have revolutionized research in immunological diseases. Co-culture of organoids and immune cells can be used to model diseases, test genetic and environmental factors, and screen drugs or compounds with health-promoting or homeostasis-restoring potentials.

## Introduction

The rapid development of stem cell-based biotechnology revolutionized immunological research in the last two decades [1–3]. This overlapped with an emerging demand for human *in vitro* models to investigate the complex microenvironment of immune cells in pathological conditions, such as cancer, infections, and inflammation. Immortalized human cell culture models failed to include the donor’s heterogeneous genetic background, the tissue structure (spatiality), the various cell composition, and the extracellular matrix interactions. On the contrary, organoids, 3D cellular structures derived from stem cells, were able to provide gap-filling tissue modelling solutions and also opened new opportunities for personalized therapies.

Organoids are capable of self-renewal, self-organization, and differentiation into various cell types resembling the organs they model. Unlike *ex vivo* organ culture of tissue biopsies or resected tissue that are rather short lived due to the lack of blood supply, organoids allow *in vitro* longitudinal studies. Indeed, organoids can be maintained in culture and expanded for months at a time, preventing the need to obtain more tissue samples from patients. There are two major classes of organoids depending on the origin of the stem cells they were cultured from adult stem cell (aSC)- and pluripotent stem cell (PSC)-derived organoids [4, 5]. ASC-derived organoids can be generated from healthy and patient tissue samples of fast-renewing tissues, e.g. gastrointestinal epithelium, and skin epidermis. ASC-derived organoids preserve the genetic and epigenetic background of their origin, and they can contain specific cell types that were not possible to culture *in vitro* before (e.g. Paneth cells, enteroendocrine cells) [6]. Thus, aSC-derived organoids have become high-potential tools for both disease modelling and precision medicine research. PSC-derived organoids can be cultured from embryonic or induced pluripotent stem cells (ePSCs and iPSCs, respectively). Due to their generation process, iPSC-derived organoid cultures can develop, on the one hand, cells of two cell types allowing researchers to study interactions between epithelial and mesenchyme cells. On the other hand, iPSC-derived organoids contain less patient-specific features [7]. Organoids are typically grown in 3D cultures preserving many features of the original tissue they are modelling. Organoid-derived cells can also be grown in 2D monolayers providing interacting cell surfaces for co-cultured immune cells, e.g. T cells, macrophages, and innate lymphoid cells [7].

In addition, light has been shed on mechanical factors essential to lead to full maturation and differentiation of stem cell-derived progenies. In systems that do not recapitulate at least part of these factors (e.g. shear fluid forces, cell monolayer stretches to mimic peristalsis), stem cell-derived cell types display more foetal-like than mature cell type features. Therefore, this drastically affects the response observed to any tested condition. Furthermore, previous studies have shown that the presence of other types of cells, such as immune cells with organoid cultures, is instrumental for certain organoid cells to differentiate and function correctly [8, 9]. Re-creating a more controlled environment or set of environments *in vitro* has become instrumental to achieving more accurate modelling of the organ under study and deciphering the mechanisms behind these cell–cell interactions. For that, it is essential to enable the mature behaviour of organoid-derived cells by combining stem cell-derived models and other cells within microfluidics systems [10].

**Figure 1.**
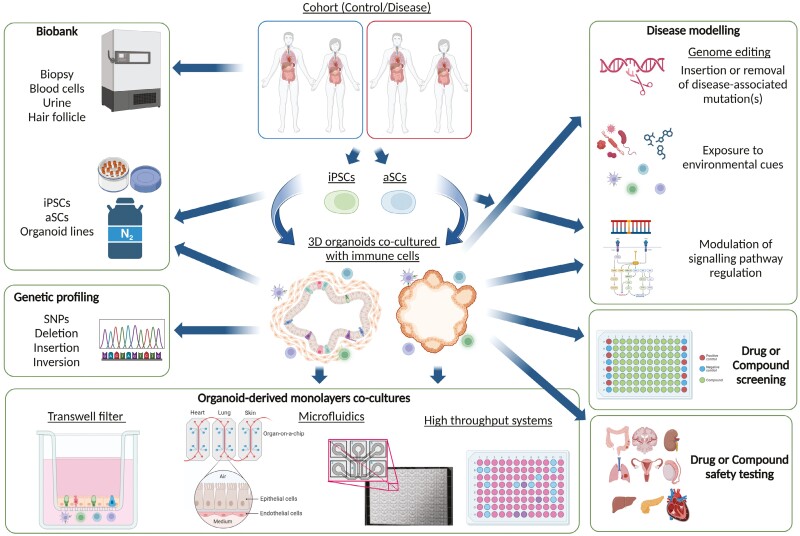
Potential use and translational applications of patient-derived organoids. 3D organoids derived from diseased or control patients can be co-cultured as 3D and monolayers with other cell types, such as immune cells. Such models have revolutionized research in immunological diseases. Tissue, stem cells and organoid lines can be collected in biobanks and shared between organoid research hubs. To increase the accuracy of these *in vitro* models, organoid-derived cells can also be grown in complex but low-throughput or simple microfluidic and high-throughput systems. As such, they can be used to model diseases, test genetic and environmental factors and screen drugs or compounds with health-promoting or homeostasis-restoring potentials.

In this review, we first summarize the most recent advancements in disease modelling with organoid and immune cell co-cultures. Then, we introduce the benefits of organ-on-chip models, a cutting-edge microfluidics-based technology that allows even more precise manipulation of the cells’ environment, including oxygen level, liquid flow or mechanical movements (Fig. 1). Finally, we highlight possible future applications and required infrastructures for immune research with organoid in personalized therapy developments, which holds high promises for treating yet incurable diseases, e.g. cystic fibrosis, inflammatory bowel disease, or cancer.

## Co-culturing immune cells with organoids to model diseases

As organoids preserve their donors’ genetic background and resemble cellular composition partially or fully of their original tissue, they possess huge potential in modelling healthy and disease tissue microenvironments. Therefore organoids provide a versatile system for immunological research to investigate the complex interactions of immune cells and the tissue in which they function. Co-culturing organoids and immune cells remains nonetheless challenging: it demands to satisfy both systems’ requirements in the culture medium, which in some cases, e.g. iPSC-derived kidney organoid—T-cells co-culture may not be compatible because of cross-reaction to components in the other system’s medium [11].

Another challenging step is to choose and prepare the appropriate organoids and immune cells. Depending on the aim of the experiment, co-cultures can be allogeneic and autologous if the donors of the immune cells and organoids are different or the same, respectively. Multiple sample collection from a single donor can still be challenging and requires more skill sets as well as ethical and legal preparations. Solving these issues motivated researchers to isolate immune cells from the same biopsy sample that is used for organoid generation [12]. Transgenic mouse models provide a broad toolset for co-culture systems, which can be utilized for further applications to human research [13, 14].

The rapid emergence of other cutting-edge technologies in sequencing, imaging, mass spectrometry, and bioinformatics further supports the thorough analysis of organoid-based models. Here, we shortly present three interconnected fields that have successfully been using organoids with promises of major breakthroughs in developing personalized therapies in tumour immunology, inflammation, and tissue regeneration.

### Modelling tumour immunology with organoids

Tumours consist of neoplastic and non-neoplastic host components, termed tumour microenvironment (TME). TME has a complex structure and unique immunological milieu, including NK cells, macrophages, dendritic cells, lymphocytes, and myeloid-derived suppressor cells [15]. As TME modulates cancer progression and drug response, understanding cell–cell interactions in a tumour is crucial for therapy improvement. Tumour-derived organoids (tumouroids) provide a unique platform for TME modelling: various oxygen and nutrient (gradient) availability, immune suppressive core, interaction with the extracellular matrix, and also the immunological environment by co-culturing with immune cells [16].

Although establishing human organoid-immune cell co-cultures is challenging, several methods have already been developed in the field. One of the major challenges is the application of immune cells to tumour organoids from the same donor. One of the solutions for this obstacle is to co-culture autologous peripheral blood mononuclear cells (PBMCs) with patient-derived tumour organoids. Dijkstra et al. established a co-culturing system with tumour organoids derived from colorectal cancer (CRC) or non-small cell lung cancer (NSCLC) tumours and peripheral blood lymphocytes. This platform was successfully used for assessing the efficiency of T-cell mediated tumour cell killing [17].

Neal et al. developed an alternative, air–liquid interface co-culturing system: where the tumour organoids and immune cells were grown from minced primary tumour samples embedded in a collagen block. This special setup recreates the original tumour environment by allowing the growth of other cell types: myofibroblasts and several immune cells. They established 19 human tumour-organoid cultures, including kidney, pancreas and lung tumours. They showed that the culture’s tumour infiltrating lymphocytes (TILs) can preserve the original tumour-resident lymphocytes’ T-cell receptor repertoire and can be activated by checkpoint inhibitors (anti-PD1, anti-PDL1) to enhance anti-tumour cytotoxicity [18]. As this system can model the TME very well, it enables the modelling and monitoring of immune suppression and drug testing *in vitro*.

A great challenge of this technique is to preserve the T-cell repertoire with increasing organoid passaging, which is required for expanding the cultures for high-throughput drug testing. Murine organoid models often facilitate the subsequent development of novel technologies based on human organoid cultures. For example, the platform by Zhou et al. applies a two-step murine pancreatic tumour organoid-T-cell co-culture system to screen drug candidates. This platform involved the modelling of the immunosuppressive TME during drug screening of the co-cultures, which allowed the identification of the most potent drugs [19]. A combined effort of the field will establish these technologies as part of the clinical routine decision-making for choosing the right therapy for the patients in the future [20].

### Modelling inflammation with organoids

Inflammation is a complex immunological process that involves the recruitment of immune cells to the site of infection or tissue damage to eliminate the cause of inflammation and restore homeostasis. Although short-term inflammation is beneficial for the body, either local or systemic chronic inflammation can lead to severe pathological conditions: cardiovascular diseases, neurodegenerative diseases, and inflammatory bowel disease (IBD) [21]. Modelling these inflammatory diseases with patient-derived organoids can uncover molecular mechanisms underlying the pathologies.

In this review, we use IBD as an example to show how organoids can be exploited to model and investigate complex inflammatory diseases. IBD is a multifactorial disease that can affect several parts of the gut (Crohn’s disease, CD) or is restricted to the colon (ulcerative colitis, UC). Although both genetic and environmental factors have been shown to contribute to disease development, the underlying mechanisms have not been fully understood yet. We can observe malfunctioning epithelium (reduced mucus layer, defects in barrier function) and immune response (enhanced recruitment of T-cells, proinflammatory cytokine production) with dysbiotic gut microbiome [22]. Most of these aspects of IBD can be modelled by organoids.

A simple but more widespread modelling of inflammation is supplementing the organoids’ medium with one or a cocktail of pro-inflammatory cytokines. This experimental setup can directly study the effects of cytokine on the organoid cells without any further modification of the culture medium, which might be necessary in case of co-culturing systems. In a recent paper, Pavlidis et al. investigated the role of the cytokine IL-22 in UC pathogenesis. Mapping the transcriptional landscape of IL-22-treated colonoids by computational biology tools revealed that the IL-22-regulated genes were enriched with CXCR2 + neutrophil chemotaxis controlling genes. This suggests that IL-22 has a pronounced role in recruiting CXCR2 + neutrophils to the colon in UC patients [23]. In another study, the same group further expanded their investigation by mapping the transcriptional landscape of additional immune-modulating cytokine-treated human colonoids. Using cutting-edge integrated systems biology tools they uncovered that UC patients with similar macroscopic inflammation can have significantly different cytokine-responsive transcript profiles, which well predicts the responsiveness to anti-cytokine therapies [24]. Analysing patient-derived organoids with omics and systems biology approaches can lay the basis for successful personalized therapy development in the future.

IBD can also be modelled by organoid-immune cell co-culture systems. Takashima et al. investigated the interactions between human (CD4 + and CD8+) T-cells and human colon epithelial cells in organoids. They demonstrated that T-cells can induce cell death in both allogeneic and autologous colonic organoids *via* IFN-γ, a pro-inflammatory cytokine. Furthermore, they showed that neutralizing antibodies against IFN-γ protected the organoids from elimination. This study demonstrated *in vitro* that dysregulated T-cells could cause epithelial injury, underscoring the importance of this cell–cell interaction in IBD patients [25].

### Modelling tissue regeneration with organoids

Immune cells are prominently known for identifying and eliminating infections and tumour cells. But, immune cells also have a highly important role in non-immune processes such as tissue regeneration, wound healing, and promotion of organ development [7]. Tissue regeneration is a complex process that involves proliferation and differentiation of progenitor/stem cells. ASC-derived organoids, which contain mostly stem cells that can be induced to differentiate, provide a perfect platform to study both proliferation and differentiation. The proliferative capacity of organoid stem cells can be measured by the size of 3D organoids, while the differentiation can be characterized by identifying matured cell types with specific immunolabeling techniques or single-cell sequencing.

Organoid medium supplementation experiments revealed that cytokines produced by immune cells are crucial in tissue homeostasis and regeneration. The multifaceted IL-22, besides its immunomodulatory effects [23], was also shown to play a crucial role in epithelial homeostasis and regeneration by inducing intestinal stem cell proliferation *via* STAT3 [14]. Another cytokine, IL-27, was shown to restore epithelial barrier function after inflammation in human colonic 2D monolayers [26]. Such studies with cytokine cocktail treatments will be able to better mimic the inflammatory cytokine environment and reveal its molecular impact on all participating cells (tissue and immune cells).

Biton *et al*. investigated the role of T helper (Th) and Treg cells in the maintenance of the intestinal stem cell (ISC) pool in mice. They found, with both co-culture and cytokine supplementation experiments, that CD4^+^ Th cells or IL-13, IL-17 treatment leads to the reduction of the ISC pool and the increase in cell differentiation, while the effect of Treg cells or IL-10 is the opposite, they favour for stem cell pool expansion [13]. Furthermore, Biton *et al*. exposed the complex relationship between epithelial and immune cells. They revealed that ISC can express MHC-II molecules on their surface and function as non-conventional antigen-producing cells, activating the CD4^+^ Th cells [13, 27]. Later this finding was confirmed in human studies and has opened new investigations to identify further roles of the MHC-II expressing epithelial cells in obesity, IBD, and cancer [27, 28].

In summary, organoids proved themselves to be a powerful tool in immunological research and disease modelling. They offer a novel opportunity to extract high-dimensional molecular data capturing intercellular interactions through approaches such as single-cell transcriptomics, spatial omics, and high-content imaging. Medium supplementation experiments can shed light on yet unknown effects of a specific cytokine on different cell types of a tissue. Although the establishment of organoid-immune cell co-cultures is challenging, it gives a deeper insight into the bi-directional interactions between the immune cells and other cell types during their migration or function.

While organoid culturing needs specific training, it does not require more resources than a tissue culture facility. Also, these models offer an adaptable high throughput capability for screening drugs or other signalling compounds in 96- or 384-well plate formats. Accordingly, organoids have already been applied in Pharma Research and Development departments. However, in the context of co-cultures with immune cells for mechanistic studies and high-resolution readouts, they still have limitations in their applicability e.g. compatibility of organoid and immune cell culture medium, the number of cytokines or cytokine cocktails that can be studied at the same time. In Section 3, we will present microfluidic-based platforms, which have brought solutions to some of these issues, introduced new features such as physiological shear flow and mechanical stimuli, and extended the applicability of organoid technologies toward mechanistic research, personalized therapy development and high-throughput drug screening.

## Platforms facilitating large-scale analysis of immune-organoid systems

**Figure 2.**
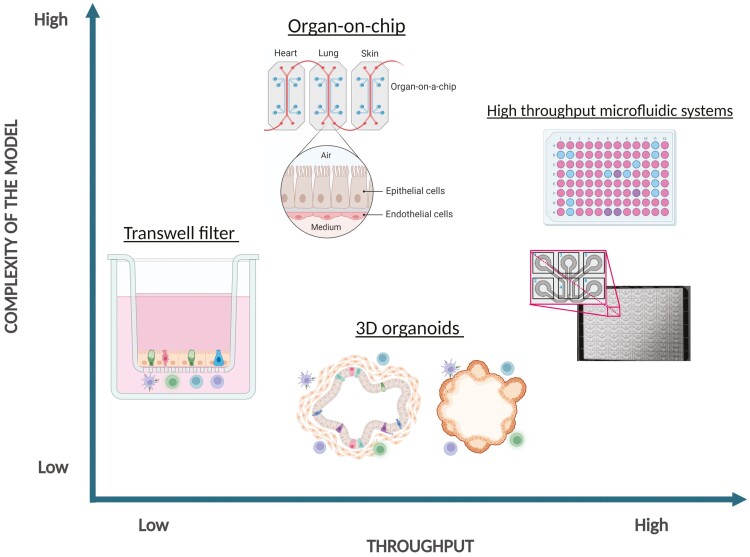
Complexity versus throughput of organoid-immune co-culture systems. Recapitulating organ-mimicking complexity in *in vitro* models and applying it to high-throughput platforms remains challenging. Massive scientific efforts invested in the field of bioengineering and microfluidics will see the development of further improved systems in the near future. Until then, the choice between model high complexity and high-throughput lies in the exact scientific questions scientists want to ask. Complexity and high-throughput systems are not mutually exclusive and will complement each other before they can be combined in future technology developments. Created with BioRender.com.

Microfluidic systems started to be developed around the same time as stem cell-derived organoid models. Most systems provide fluidics, sensors, and pumps that apply mechanical and shear forces to cultured cells, reproducing, for example, luminal transit and peristalsis of the normal gut in a highly controlled manner. The addition of shear forces from both flow and peristalsis was shown to fasten the maturation of epithelial monolayer in culture and to increase the similarity with *in vivo*-observed functions. Examples of these observations include barrier and absorption functions obtained after 5 days of culture (instead of 21 days in static Transwell models); intestinal microvilli and glycocalyx layer after only 4 days of culture; and boost of cell metabolism [29, 30]). Microfluidic systems often provide the juxtaposition of different compartments, facilitating the co-culture of different cells (e.g. immune cells with epithelial cells) and fine-tuning compartment-specific triggers. Many systems comprise at least two compartments, separated by a semi-permeable support membrane that allows the culture of cell monolayers (e.g. epithelial cells; endothelial cells; Fig. 2). The upper and lower channels could also be the site of co-culture with other cell types (e.g. immune cells) besides bringing medium (e.g. nutrients, oxygen). Others are capillary-based systems interconnected with microchannels to test the impact of signalling molecules and vasculature (Table 1). In the last decade, the combination of constantly evolving microfluidic systems with organoids has revolutionized and broadened their range of research applications, particularly for medical and pharmaceutical purposes [51–53]. With more compartmentalized environments, more diverse actors (e.g. cells or physiological conditions) can be included increasing the complexity of the system [54–56].

**Table 1.** Examples of microfluidic-chip systems that can be used for co-culture with immune cells or other cells in more physiologically relevant environments to study cell-cell interactions

Chip systems	Company	Organ modelled	Already used for organoid- or iPSCs-derived cells (Y/N) and examples of co-cultured cells	Links and references
Organ-on-a-chip	Emulate	Colon Duodenum Lungs Brain Kidney Liver	Y e.g. human type 1 and 2 lung alveolar epithelial cells, microvascular endothelial cells with CD14^+^ monocytes, CD3^+^ T cells, and CD19^+^ B cells, epithelial cells, endothelial cells, neutrophils, microbes neurons, glial cells, microglia, astrocytes, pericyte, endothelial cells	https://emulatebio.com/organ-chips/ [31–33]
Organoplate	Mimetas	Pancreatic tumour Intestine Liver	Y e.g. Pancreatic ductal adenocarcinoma organoids with pancreatic stellate cells; hepatic organoids and endothelial cells	https://www.mimetas.com/en/products/ [34–36]
Biomimetic environment on chip	BEOnChip	Blood brain barrier (BBB) gut Skin Lung	Y e.g. keratinocytes and dermal fibroblasts	https://beonchip.com/technology/ [37]
inCHIPit, comPLATE and MUSbit	Bi/Ond	Muscle vascularization Kidney organoid vascularization	Y e.g. cardiac muscle cells and endothelial cells Kidney organoids and human umbilical vein endothelial cells	https://www.gobiond.com/ [38]
IC-CHIP	Initio cell	Lung Liver Brain Gut Vasculature Bladder cancer	Y e.g. Breast cancer cells and macrophages	https://www.initiocell.com/ [39]
SynBBB, SynRAM, SynTumor, SynALI, SynTox, microfluidic chips	Synvivo	Vasculature Lungs Liver Blood Brain Barrier	N e.g. endothelial cells with astrocytes, pericytes and neurons	https://www.synvivobio.com/ [40]
HuMIX	Luxembourg University	Intestinal epithelium, immune system and microbiota	N e.g. intestinal epithelial cells such as Caco-2 cells with patient-derived CD4^+^ T lymphocytes	https://www.fnr.lu/research-with-impact-fnr-highlight/poc-pocket-sized-intestines-the-humix-model-enables-intestinal-flora-to-be-investigated-under-real-conditions/ [41]
HUMIMIC	TissUse	Multi-organ Chips: Brain, lung, heart, Hair follicle, lymph node, intestine, pancreas, kidney, liver, bone marrow, vasculature, adipose tissue, skin, thyroid	Y e.g. Liver or kidney organoids with mesenchymal stromal cell-derived small extracellular vesicles iPSC-derived liver and brain spheroids	https://www.tissuse.com/en/ [42–46]
The human-on-chip (service company, that does not sell devices)	Hesperos	Multi-organ chips Liver Muscle Heart Pre-neurons Kidney Monocytic cells	Y e.g. Biopsy-derived hepatocytes, iPSC-derived cardiomyocytes and arm or leg skeletal muscle-derived myoblasts	https://hesperosinc.com/ [47–50]

Many chip systems have been described in the literature, yet, only a few are commercially available [57, 58]. Nonetheless, several startups and well-established biotech companies now propose a versatile range of microfluidics and sensors that can be adapted to existing or novel organ-on-chip or tumour-on-chip systems. The use of microfluidics increases the systems’ output alongside the complexity of cellular assemblies mimicking one or multiple organs to study intra- and inter-organs signalling. Some have already been applied to organoid-derived cell cultures, and others have been used mostly for single-cell and tumouroid investigations (Fig. 2; Table 1). The number of organs/tissues modelled by organoids applied to organ-on-chip systems is constantly rising. This has already permitted, for example, the co-culture of peripheral blood mononuclear cells (PBMCs) with patient-derived kidney organoids in an immune cell-activating environment (IL-2) [42]. Using a similar microfluidic system intestinal epithelial cells were co-cultured on chips with infiltrating neutrophils to model tissue inflammation [59]. In another study, primary lung alveolar epithelial and endothelial cells were co-cultured in Emulate chips with T and B lymphocytes as well as monocytes to look at immune response following viral infection [31]. Lung-on-chip as well as liver-on-chip models have allowed the culture of alveolar or bronchiolar epithelial cells, as well as hepatocytes, in co-culture with other cells such as immune cells [60–62].

Distant organ interaction axes can now be interrogated using chip and microfluidic systems. Multi-organ-on-chip systems, linking several organ-specific chips, now allow studying distant organs crosstalk (e.g. gut–brain, intestine–liver, liver–heart, liver kidney axes, Fig. 2) [43,44,47,63–65].

The combination of chips, microfluidics, and organoid-derived cells presents the advantage of studying the cellular response of disease-specific patients to drugs or immune cells or microbial compounds [42, 66]. A subset of systems now come in high-throughput format [34, 67], enabling to screen co-culture of the same cells with cells from different tissues, signalling compounds, or drugs (e.g. T cells, macrophages, neurons; cytokines, biologics [68–71]). Reciprocally, these systems can be used to screen the response of cells derived from different organoid lines or different donors exposed to the same challenge, illustrating patient-to-patient variability (and the need to move towards personalized medicine—Table 2). It is now accepted that these systems can and should be used as pre-clinical risk assessment tools as they provide various physiologically relevant ways of interrogating patient-, disease-, tissue-, and challenge-specific responses of the organ/tissue of interest. For example, *in vitro* models using endothelial cell-lined organ-on-chip and human-derived blood have allowed us to test and predict the risk of thrombosis, a life-threatening side effect of some immuno-modulatory treatment of autoimmune diseases [81]. Where this had not been permitted before due to the lack of relevant models, organ-on-chip systems allowed testing the impact of such anticoagulant drugs on endothelial activation, platelet adhesion, platelet aggregation, fibrin clot formation, accelerating the determination of thrombosis risk-associated with novel drug development. Similarly, blood–brain barrier models on a chip using iPSCs derived from patients with neurological diseases permitted the test of blood-brain barrier permeability of pharmacologic and their cytotoxicity on neurons in patients’ vasculature, showing disease-specific lack of transporters and disruption of barrier integrity [82]

**Table 2.** Major advantages of using microfluidic systems to study cell–cell interactions (e.g. epithelial cell immune cell interactions)

Advantages	Reference(s)
Greater capacity to recreate more accurate and reproducible tissue/organ architecture	[56, 72, 73]
Compartmentalized environments	[74]
Prevention of organoid fusion while permitting organoid–organoid communication	[75]
Access to the luminal side of the epithelial layer (co-culture with microbes or microbial compounds enabled; readouts broadened), and rare, specialized cell types seldomly found in conventional organoids.	[41]
Extended life-span (up to months)	[76]
Organoid–organoid co-culture, combined with function sensors to look at organoid–organoid and cell–cell interactions between different tissues or organs	[77]
Gap-bridging between animal models and clinical trials	[78]
Drug screening, patient-specific modelling, regenerative medicine	[51, 79, 80]

However, if all organ-on-chip systems have brought greater recapitulation of organ physiology and cellular functions to *in vitro* models, these systems are not universal and remain to be improved and adapted to model specific tissue, disease, and cell type (see Table 3). Indeed, the protocols used to differentiate organoid-derived cells vary considerably between laboratories, affecting the reproducibility expected with such systems. Also, not all chips and microfluidic systems will work for all organ/tissue modelling, and the chip material composition, the fluidics control systems, and many other parameters need to be optimized for each model [83, 93]. For example, media compatibility between the different co-cultured cell types has to be assessed first and might require novel media development prior to co-culture being possible. All cell types might be functional in the same medium [31], or they both need slightly different media as factors essential in one medium could influence and affect the functions of the other, as shown for the co-culture of kidney tubuloids and T helper cells [11]. Furthermore, human iPSC-derived organoids do not produce fully mature cells, even in microfluidics systems. Different approaches are currently being developed to circumvent this technical issue, such as electromechanical stimulation, overexpressing maturation-related microRNAs, introducing growth hormones, and increasing culture time [94–97]. The need to scale up these systems to accommodate high-throughput readouts for drug/compound screening depends on the automatability of the systems [92]. It is, therefore, essential to bear in mind that these more complex *in vitro* co-culture systems remain simplistic compared to a whole organ and that additional development will always contribute to closer modelling of the *in vivo* situation. Including technical and biological replicates for each system, the number of treatments tested in parallel, the number of readouts and the requirements for each downstream method as well as the overall cost/affordability, will determine the size and feasibility of each experiment. Whether comparing 4 or 32 conditions, each system, model and research group will have to make the appropriate compromises to make each experiment meaningful. Integration of many data levels obtained for each system will require access to high-power computational resources to understand the sophistication of cell–cell interactions [98].

**Table 3:** Major limitations in using chips and microfluidics for the culture of organoids and for their co-culture with other cells

Limitations	Reference(s)	Strategy for decreasing the limitations
No harmonized differentiation protocols between labs due to this fast-moving field.	[83]	Establishing standardized protocols and defining reference compounds and biomarkers can be achieved by a collaborative effort of the experts of the field or the organoid/stem cell facilities like CorEuStem COST action.
No standardized reference compounds or biomarkers available yet	[78]	
iPSCs, even on organs-on-chip systems, are typically immature/foetal-like phenotypes	[84]	The limits of the given model should be always considered before their application. More fundamental studies are required on how to mimic the microenvironment of the different cell types to support their differentiation and functions *in vitro*.
Immune cells are added to the system, therefore not reflecting their *in vivo* recruitment or tissue physiology	[85]	
Restriction in the choice of chip material, with commonly used material (e.g. polydimethylsiloxane (PDMS)) absorbing small hydrophobic compounds, or shrinking	[86, 87]	Encouraging collaborations between engineers, material scientists and biomedical researchers to extend the borders of chip applications. New funding opportunities of chip technology development, will lead to improved biosensors and scalability and also introduction of novel membrane materials.
Very few systems offer a high-throughput format; most not available being commercially	[88–91]	
Need for scalable (higher through-put) and automatable sensors for monitoring the physiological state and transient response of the cell monolayer and run parallel experiment reproducibly	[92]	

The output information obtained in co-culture with immune cells is also limited by the technical challenge of cultivating primary immune cells. It is not possible yet to reproduce the diversity and dynamic nature of immune cells *in vitro* as it is found *in vivo*. No culture system yet can mimic the education path immune cells take inside the body, nor their exact recruitment process before reaching their effector site. Co-cultures of organoids with immune cells require the isolation of immune cells directly from tissues, which makes rare or specific populations of immune cells challenging to study.

Many of the technical limitations listed in Table 3 are currently being addressed to improve how microfluidics can support the co-culture of organoid-derived cells with other cells, such as immune cells. Huge efforts in bioengineering research and industrial R&D are being invested in adapting further existing platforms or developing new platforms with better sensors, fluidics, and biomaterials. In addition, chip systems connected to single-cell omics readouts, including spatial transcriptomics, metabolomics, and lipidomics, are increasing the resolution of datasets. Such approaches will increase the scale of the experiments and will facilitate the understanding of what specific immune cells do in their interactions with other cells. Finally, the research based on organoid and co-culture systems is pursued in isolated networks, consortia, and hubs, taking place in different locations or research fields. More crosstalk and collaborative efforts must be placed in international research group interactions to facilitate access to these sophisticated *in vitro* models.

## Future perspectives

### Disease modelling with genome editing techniques

Genetic variations and mutations are crucially important in disease modelling and can be examined *in vitro* using organoids. Deriving organoid lines from patients carrying one or a combination of disease-associated genetic mutations has proven highly relevant in understanding the contributions of the genetic background to disease development [99, 100]. It is vital to understand how these genetic variants can influence the response to or from the immune system in diseases [101, 102]. To study the role of disease-associated genetic variants, applying sophisticated gene-editing technologies such as CRISPR-mediated knock-out or knock-in is a popular approach. This allows the introduction of gene modifications (e.g. the introduction of a mutated variant or the replacement of a mutation with the reference genotype) at a much higher frequency and with significantly shorter preparation time than previously used techniques [103]. Since its development, CRISPR technology has been extensively improved and is now proven to be well-superior to other approaches [104–106]. The conventional CRISPR/Cas9-based technology tended to introduce other off-target, potentially deleterious, double-strand breaks that prevented the reliable use of this genome editing technique in clinical repair. Recently, modified Cas9 enzyme base editors were developed that specifically aid the conversion of C-G to T-A base changes (CBE) or A-T to G-C (ABE) at the target site and prevent the most off-target double breaks, making this gene editing procedure much more robust and reliable for clinical applications [107]. CRISPR-based base editing protocols have since been applied to genetically modifying stem cell-derived human organoids to model various diseases [107–109] affecting organs such as the liver and intestines [110, 111], kidneys [112, 113], or pancreas [114].

Subsequent phenotypic functional assays or omics approaches can then be used to validate the causal effect of the genetic modification on the observed phenotypes (e.g. immune-epithelial cell interactions). This also permits screening drugs [115] or compounds of interest that could restore a normal phenotype or dampen the disease-associated dysfunction [116–118].

### Patient-derived organoids: the perfect model to develop personalized therapies

The pathogenesis of many pathologies has now clearly been described as multifactorial, combining genetic predisposition, environmental triggers, dysregulated immune response, microbiota, and lifestyle to evolve towards a disease onset [119–121]. Access to patient cohorts, hence to disease-specific as well as control tissue samples, and related clinical data, in parallel to the wide range of readouts now available, create the perfect research landscape for disease modelling and translational research. To obtain meaningful screening data that can subsequently inform the diagnosis or treatment choice of disease-specific patients in clinical settings, academic and pharma industry research groups have initiated generating organoid line resources in the form of organoid biobanks. These groups are becoming hubs that exchange knowledge, share methodologies, and provide a much more robust and harmonized framework for collaborative mechanistic and translational research. In combination with patient-derived organoid models, such large projects can help recapitulate patient-to-patient variability, patient-specific genetic contributing factors (knock-in, or -out, of SNPs and other mutations into organoid lines, see section 4.1), interactions with other host cells (e.g. immune cells), and selected microbial or metabolic challenges identified as contributing factors to the disease or altered drug response [122–124]. Such complex systems will enable scientists and clinicians to comprehend why gut homeostatic functions are dysregulated in certain patients and why certain treatments will not work in patients, although they display the same symptoms.

It is important, however, to consider certain criteria before embarking on such investigations:

The number of organoid lines representing each specific patient group will need to be carefully discussed with statisticians [125, 126]. Power calculation will need to be checked for each scientific question to consider the type and precision of the measurements planned, how they vary between samples in each group, how big a difference will be considered to have clinical significance, what type of statistical tests will be used etc. A lot of time will therefore be necessary for the project preparation.

If the project aims at co-culturing organoid-derived monolayers with immune cells or other cell types, those cells will have to be obtained ideally from the same patient. The co-culture of autologous cells can reflect more accurately the true interactions that take place in the individual. Indeed, the co-culture of organoids with immune cells from a different donor, although possible, will lead to a potent immune response due to HLA mismatch. This will mask the specific response expected from autologous epithelial and immune cell interactions [127]. Autologous immune cells can be obtained in different ways. Immune cells can be obtained from the same patient’s blood at the time when the intestinal biopsies are taken. This will, however, be ethically challenging to justify such additional sampling for research purposes only, and it will require further patient-consenting procedures [128]. Peripheral blood cells are, however, not the same as tissue-resident cells and will not behave exactly as resident cells would, introducing another source of data variation in the results. Tissue-resident immune cells are, unfortunately, rare and tricky to isolate in high enough numbers for co-culture experiments [129, 130]. Furthermore, immune cells are short-lived cells, and if isolated at the same time as the biopsy-derived stem cells required for creating and expanding organoid lines, they will no longer be in the state they were in when isolated from the individual.

Some of the cells needed for co-culture with the organoid-derived monolayers cannot be isolated from tissue or blood patient samples. To circumvent this issue, those cells must be generated from cells obtained from the same individual. These cells from hair follicles, skin, saliva, or urine samples are then reprogrammed *in vitro* into induced pluripotent stem cells that can then be used to generate the type of cells to be co-cultured with organoids [131]. This complicates and considerably lengthens the generation of all samples needed for such a project, impacting the feasibility, delivery of the objectives and, consequently, the sample size, requiring revisiting the power calculation. This could be a strong bottleneck in the design of such studies.

Beyond the mechanistic understanding of certain pathologies and the development of novel or repurposing of existing drugs to treat them, clinical research is also evolving in the field of patient-specific tissue regeneration using organoid self-grafts. After deriving organoids from patient tissue and identifying the mutation(s) altering important organ functions involved in the pathology, one can edit the genome of these organoids to either block an exacerbated function responsible for the malfunction of the tissue or restore the correct genotype in the stem cells of the organoids, as explained previously [112, 114]. Once modified and tested *in vitro*, those organoids can then be transplanted back into the patient of origin [132, 133].

### Organoid facilities and biobanks

As the versatile potentials of the organoid technology became apparent, more and more organoid facilities were founded worldwide. The common aim of these facilities is to support organoid researchers exploring the novel applications of this young technology. Most organoid facilities are working in close collaboration with clinicians and hospitals (NIHR Imperial BRC Organoid Facility at the Imperial Healthcare Hospitals) and often are integrated into universities or research institutes (e.g. Gut-HOP, Centre for Host-Microbiome Interactions at King’s College London, UK; Karolinska Stem Cell Organoids (KISCO), Sweden; BIH Core Unit pluripotent Stem Cells and Organoids (CUSCO), Germany). Some facilities focus on a disease or disease group, such as cancer or inflammatory bowel disease (IBD) (e.g. UCL Organoid Platform, UK; The Human Organoid Innovation Hub (HOIH), University of Calgary, Canada, respectively), while others provide support for modelling a range of diseases (e.g. The Organoid and Tissue Modeling Shared Resource (OTMSR), University of Colorado, USA). The organoid facilities usually closely collaborate with other core facilities, e.g. imaging core facilities (e.g. Organoid Core Facility, Beth Israel Deaconess Medical Center, USA), while some facilities have integrated these cutting-edge technologies, and provide platforms for organoid gene editing or high-throughput drug screening (e.g. Pluripotent Stem Cell Facility, Center for Stem Cell & Organoid Medicine (CuSTOM), USA and National Facility for Genome Engineering, Human Technopole, Italy; Organoid Shared Resource, CSHL Cancer Center, USA, respectively).

Recently a European Union funded COST Action was formed, called CorEuStem (#CA20140), to build a network of stem cell and organoid facilities to facilitate knowledge exchange among the facilities. The action aims to standardize methodologies to increase the reproducibility of organoid-related techniques, which can be a challenge due to the high variability among the organoid donors. Such effort is crucial to lay the foundations for further applications of organoids, such as personalized therapy development.

Many organoid facilities establish their organoid and/or stem cell biobank from the control and patient samples they handle. Some are available only for researchers of the hosting university or institute. But there are facilities that make their organoid lines available for commercial purchase on ATCC (e.g. Organoids (ProjectGro), Wellcome Sanger Institute, UK), or from them directly (e.g. HUB Organoids, the Netherlands). These biobanks will further facilitate both fundamental and drug research by providing a heterogeneous patient (and control) organoid and/or stem cell collection for targeted studies.

It is important to bear in mind that patient-to-patient variations in any human organoid data are inherent due to genetics and epigenetic regulation mechanisms that retrace the lifestyle and medical history of each individual. Studies involving disease and control patient cohorts have enabled the following of time-dependent responses to specific or multiple exposures and to observe how different sub-groups of patients behave similarly [134].

Many chronic diseases illustrate the strong variability in drug response that exists between patients suffering from the same disease and having similar symptoms. Ethically collecting the diverse patient responses (multi-omics readouts) along with clinical metadata can allow classifying different profile clusters or patient groups based on their coding or non-coding single nucleotide polymorphism (SNPs) [99, 135]. Organoids can be derived from many representative patients of each group or cluster within a cohort, and the response diversity observed can be validated and deciphered in these *in vitro* models for translational research purposes (e.g. drug screening or repurposing and regenerative medicine) [107, 136].

The high relevance of immune cell co-culture assays in personalized medical research raises urgent demand for establishing more biobanks that store and maintain both organoid and blood samples from the same donor (e.g. Center for Engineered Multilineage Organoids in 3D Microenvironment, Istituto Nazionale di Genetica Molecolare, Italy; Human Organoid Facility & Biobank, Institute of Cancer Research, UK). These biobanks will be extremely valuable in investigating the interaction of immune cells with tumour or other cell types, e.g. intestinal epithelial cells to uncover the differences in therapy response of cancer or IBD patients. Donor-matched sample collection in biobanks will facilitate many translational research studies, increasing the robustness of the results obtained for personalized medicine approaches [127].

### Screening of drug molecules

As described in section 3 of this review, high-throughput microfluidics systems combined with organoid or spheroid culture are a much-improved set-up for screening drugs, metabolites, and compounds for beneficial properties and for restoring healthy functions in diseased patient-derived *in vitro* models. In particular, it is now much easier to co-culture organoid-derived monolayers with relevant immune cells, such as monocytic cells [9, 72]. Scalable and automatable systems are emerging, and many systems are likely to be made available in the next decades that will revolutionize the field of drug discovery and screening [137, 138]. Already several systems exist that allow vast numbers of compounds to be individually tested at one time [139]. Other systems can test combinations of drugs or dynamic sequences of drug administration on 3D organoids [138, 139]. Testing concentration gradients of cytokines, for example, can also be achieved using organoid- or spheroid-derived cells in organ-on-chip systems to recreate immune-epithelial cell signalling [140]. We can envisage that once such systems integrate greater organoid cellular diversity and complexity, relevant immune cells, and disease-associated patient genetics, these models can then be used for the screening of new or repurposed therapeutics [136, 141].

### Co-culture with microbes

Commensal or pathogenic microbes have a major impact on host-cell responses, including signalling responses to and from immune cells and responses to drug treatments [142, 143]. Chronic inflammatory diseases, for example, are characterized by an exacerbated response against microbes or microbial compounds. Such extreme and unresolved inflammatory host responses are a major cause of the onset of such pathologies (e.g. Crohn’s disease and ulcerative colitis) [144]. Dysbiotic composition and functions of the gut microbiota, the largest microbial community in the body, are often associated with many chronic digestive or distant pathologies such as IBD and metabolic or neurodegenerative diseases [145, 146]. It is, therefore, essential to comprehend the complex interactions that take place between the microbiota, the intestinal epithelium, and the immune system. Intestinal organoids are recognized as the ideal *in vitro* cellular models to study such crosstalks, in particular, to question whether they take place in a host-cell type-specific manner. Considering the inward polarity of 3D intestinal organoids, the need for access to both the apical side of the epithelial layer to reproduce the normal encounter of the gut lining with microbes and the basal side to integrate immune cells has boosted the troubleshooting creativity of scientists. From 3D organoid micro-injections [147], inversion of the organoid polarity (i.e. apical-out) [148], or generation of monolayers [149] and highly complex microfluidics systems [41, 150], continuous improvement of *in vitro* systems will enable co-culture of organoid-derived monolayers with gut microbes apically as well as innate or adaptive immune cells basolaterally, despite their different and sometimes opposite environmental requirements (e.g. oxygen levels). The different options of microbial co-culture with organoid systems have already been described and reviewed by us and others [72, 151–153].

## Conclusions

The application of organoids to the understanding of tissue immunity in health and disease has opened the door to an increasing number of applications. From access to large biobank patient-derived organoid lines to increased model complexity enabled in microfluidics systems in addition to high-throughput multi-chip format platforms, clinical research in immunological diseases is at the dawn of a big data-generating time. The impact of genetic variants and environmental cues on regulating homeostatic tissue functions is soon to be screened on much larger scales. Similarly, novel therapeutics and biologics can be screened in pre-clinical studies. The multidisciplinary approaches that are now applied to such *in vitro* models improve how they mimic an organ or tissue in a patient-specific manner. This consequently promises more reliable outcomes to drug testing and clinical trials, paving the way towards getting more patients in remission, thus with a better life.

## Data Availability

No unpublished data is included in our review manuscript.

## Abbreviations:

aSC: adult stem cell
CD: Crohn’s disease
CRC: colorectal cancer
ePSCs: embryonic pluripotent stem cells
IBD: inflammatory bowel disease
iPSCs: induced pluripotent stem cells
ISC: intestinal stem cell;
NSCLC: non-small cell lung cancer
PBMCs: peripheral blood mononuclear cells
PSC: pluripotent stem cell
Th: T helper
TME: tumour microenvironment; tumouroids, tumour-derived organoids
UC: ulcerative colitis.
